# Depot-Based Delivery Systems for Pro-Angiogenic Peptides: A Review

**DOI:** 10.3389/fbioe.2015.00102

**Published:** 2015-07-16

**Authors:** Amy H. Van Hove, Danielle S. W. Benoit

**Affiliations:** ^1^Department of Biomedical Engineering, University of Rochester, Rochester, NY, USA; ^2^Department of Chemical Engineering, University of Rochester, Rochester, NY, USA; ^3^Department of Biomedical Genetics, University of Rochester Medical Center, Rochester, NY, USA; ^4^Department of Orthopaedics, Center for Musculoskeletal Research, University of Rochester Medical Center, Rochester, NY, USA

**Keywords:** angiogenesis, controlled release, biomaterials, drug delivery, hydrogels, depot-based, review

## Abstract

Insufficient vascularization currently limits the size and complexity for all tissue engineering approaches. Additionally, increasing or re-initiating blood flow is the first step toward restoration of ischemic tissue homeostasis. However, no FDA-approved pro-angiogenic treatments exist, despite the many pre-clinical approaches that have been developed. The relatively small size of peptides gives advantages over protein-based treatments, specifically with respect to synthesis and stability. While many pro-angiogenic peptides have been identified and shown promising results *in vitro* and *in vivo*, the majority of biomaterials developed for pro-angiogenic drug delivery focus on protein delivery. This narrow focus limits pro-angiogenic therapeutics as peptides, similar to proteins, suffer from poor pharmacokinetics *in vivo*, necessitating the development of controlled release systems. This review discusses pro-angiogenic peptides and the biomaterials delivery systems that have been developed, or that could easily be adapted for peptide delivery, with a particular focus on depot-based delivery systems.

## Introduction

Therapeutic angiogenesis holds great potential for supporting developing engineered tissues, where insufficient vascularization limits size and complexity. Additionally, a number of ischemic tissue disorders would benefit from pro-angiogenic therapies by restoring blood flow to the tissue. However, no FDA-approved treatments exist to reproducibly enhance vascularization (Muir, 2009; Zachary and Morgan, 2011; Chu and Wang, 2012).

The field of tissue engineering has made remarkable progress in developing tissues to restore, augment, or replace the function of damaged tissues within the body. For example, engineered urethras remained functional for 6 years (Raya-Rivera et al., 2011). Similarly, tissue-engineered bladders (Atala et al., 2006) and trachea (Macchiarini et al., 2008) have been successfully implanted in humans, and remained functional at 46- and 4-month follow up exams, respectively. However, the success of engineered tissues has been limited to thin tissues, with engineering larger, more complex structures slowed by challenges associated with development of necessary vascularization to sustain growing/remodeling tissues (Atala, 2004).

$500 billion is spent each year in the United States to treat cardiovascular diseases, such as peripheral arterial and coronary heart disease (Go et al., 2014). Peripheral arterial disease affects 8.5 million Americans, and coronary heart disease is the leading cause of death in the United States, responsible for ~1 in every 6 deaths in 2010 (Go et al., 2014). Currently, treatment of peripheral arterial and coronary heart disease focuses on maximizing function of existing vasculature using vasodilators and anti-clotting agents, or through surgical interventions, such as angioplasty, stent placement, or bypass grafts, rather than encouraging development of new vasculature to support the tissue (Muir, 2009; Lloyd-Jones et al., 2010).

Diabetes affects 20.8 million people in the United States, with 15% of this population also affected by diabetic foot ulcers. Diabetic ulcers precede 84% of diabetes-related lower limb amputations and are a cause significant morbidity, making them a significant public health burden (Brem and Tomic-Canic, 2007). These wounds are resistant to healing due in part to decreased angiogenic response (Galiano et al., 2004), and improvements in ulcer healing have been obtained with repeated topical treatment with pro-angiogenic growth factors. However, becaplermin (recombinant platelet-derived growth factor, PDGF) is currently the only FDA-approved pro-angiogenic treatment for diabetic ulcers, and even with daily application, only 48% of patients exhibit complete wound closure over 20 weeks (Steed, 1995).

This review focuses on pro-angiogenic peptides and biomaterials exploited for their delivery. First, the process of angiogenesis including the critical factors and cell types involved in this process is briefly reviewed. The relative merits of pro-angiogenic peptides as compared to proteins are discussed, as are particular challenges associated with the use of peptide drugs. While many of the biomaterials that have been developed for the delivery of pro-angiogenic factors focus on delivery of large proteins, this review focuses on identifying materials that can be adapted for delivery of the many promising pro-angiogenic peptides that have been identified, as well as materials designed specifically for peptide delivery. Both natural and synthetic biomaterials are discussed, with a focus on depot-based (rather than injectable or orally delivered) biomaterials as they present advantages for pro-angiogenic applications.

## Angiogenesis

Vascularization is involved in tissue homeostasis, wound repair, tissue healing, and during the female reproductive cycle. In healthy tissue, development of new vasculature is a carefully orchestrated process controlled by growth factor signals. New vasculature within the body is formed by three processes: vasculogenesis, angiogenesis, and arteriogenesis (Heil et al., 2006). Vasculogenesis occurs early in development and gives rise to the primitive circulatory system, but does not occur during adulthood. Angiogenesis and arteriogenesis, however, frequently occur in adult tissue (Chu and Wang, 2012). Angiogenesis is the sprouting and growth of new, small vessels from existing vasculature, followed by the subsequent remodeling and maturation of the newly developed vasculature. Arteriogenesis typically occurs when larger arteries are occluded, and involves the remodeling of pre-existing vasculature into fully developed, functional arteries (Heil et al., 2006; Chu and Wang, 2012).

**Figure 1:**
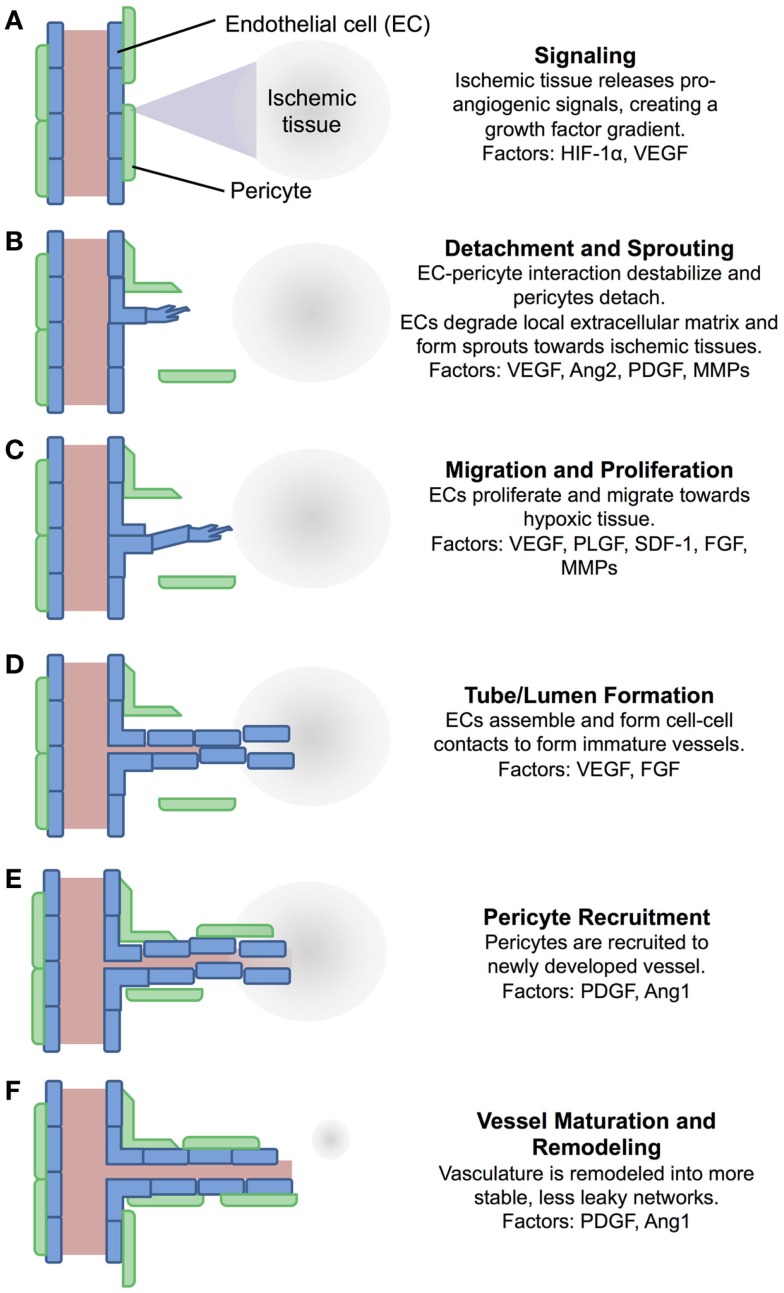
**A schematic of the process of angiogenesis**. Angiogenesis is a process tightly controlled by a number of factors. **(A)** Ischemic tissue release pro-angiogenic signals, which diffuse into nearby tissue. **(B)** Pericytes detach from nearby vessels, and ECs form sprouts. **(C)** ECs proliferate and migrate towards the signal gradient. **(D)** ECs align into immature vessels. **(E)** Pericytes are recruited to the new vessels. **(F)** Vasculature is remodeled and stabilized. Many of the factors involved in this process have been exploited for pharmacological intervention, either supplementing them for pro-angiogenic applications, or inhibiting them for anti-angiogenic applications. EC, endothelial cell; HIF-1α, hypoxia-inducible factor-1α; VEGF, vascular endothelial growth factor; Ang2, angiopoietin 2; PDGF, platelet-derived growth factor; MMPs, matrix metalloproteinases; PLGF, placenta growth factor; SDF-1, stromal cell-derived factor-1; FGF, fibroblast growth factor; Ang1, angiopoietin 1 (Ziche et al., 2004; Hirota and Semenza, 2006; Adams and Alitalo, 2007; Lieu et al., 2011; Chu and Wang, 2012; Brudno et al., 2013).

While arteriogenesis occurs in response to changes in shear stress within a vessel, angiogenesis responds to tissue hypoxia or insufficient tissue oxygen tension (Adams and Alitalo, 2007). This initiates growth factor signaling cascades that drive the formation of new vasculature toward the ischemic tissue. A schematic representation of this process is shown in Figure 1, which includes a summary of key growth factors involved in the process. First, low oxygen tension inhibits the intracellular degradation of hypoxia inducible factor-1α (HIF-1α), causing HIF-1α accumulation and allowing it to bind with HIF-1β and activate hypoxia-responsive elements within target genes (Hirota and Semenza, 2006). This causes production of a number of angiogenic growth factors, such as vascular endothelial growth factor (VEGF) (Forsythe et al., 1996), which then diffuse into the nearby tissue (Figure 1A) (Hirota and Semenza, 2006). These factors signal nearby vasculature, causing detachment of pericytes and sprouting of endothelial cells (ECs) toward the VEGF gradient (Figure 1B) (Hirota and Semenza, 2006; Adams and Alitalo, 2007). ECs then migrate in the direction of the gradient, degrading the local extracellular matrix and proliferating in response to factors, such as VEGF, fibroblast growth factor (FGF), and stromal cell-derived factor-1 (SDF-1), producing the required number of cells for vessel formation (Figure 1C) (Kuhlmann et al., 2005; Adams and Alitalo, 2007; Lieu et al., 2011). ECs align in tube-like lumen structures, forming an immature vascular network (Figure 1D). Pericytes are then recruited to the newly formed vasculature (Figure 1E) and the pericyte–EC interaction is stabilized by factors, such as PDGF and Angiopoietin 1 (Figure 1F) (Ang1) (Hirota and Semenza, 2006). A variety of growth factors are involved in this process as indicated in Figure 1, with some produced by the ischemic tissue itself and others by ECs and pericytes, often in response to previously expressed factors (Forsythe et al., 1996; Ziche et al., 2004; Kuhlmann et al., 2005; Hirota and Semenza, 2006; Adams and Alitalo, 2007; Lieu et al., 2011; Chu and Wang, 2012; Brudno et al., 2013).

Dysfunctions in angiogenesis can lead to serious pathological conditions. Excessive angiogenesis occur in diseases such as cancer, rheumatoid arthritis, age-related macular degeneration, and diabetic retinopathy, while insufficient angiogenesis is associated with diseases like coronary arterial diseases, stroke, and impaired wound healing (Ziche et al., 2004). The development and delivery of anti-angiogenic drugs are a large, exciting area of current research that has been reviewed elsewhere (Nishida et al., 2006; Folkman, 2007; Segal and Satchi-Fainaro, 2009; Welti et al., 2013; Vasudev and Reynolds, 2014). Restoring vascular homeostasis holds great potential for the treatment of ischemic tissue diseases, and as a result, has become an area of great interest in the fields of drug discovery, drug delivery, and tissue engineering (Atala, 2004; Ziche et al., 2004; Vinoth Prabhu et al., 2011; Chu and Wang, 2012). In this review, we focus on biomaterials for the delivery of pro-angiogenic drugs, with a particular focus on delivery of pro-angiogenic peptides.

## Pro-Angiogenic Therapies

Pro-angiogenic approaches include delivery of angiogenic proteins (Losordo and Dimmeler, 2004; Silva and Mooney, 2010) or gene therapy resulting in the expression of these proteins (Henry et al., 2007; Gupta et al., 2009), peptide drugs (Lane et al., 1994; Finetti et al., 2012), a limited number of small molecule drugs (Wieghaus et al., 2008), as well as cell-based approaches (Rustad et al., 2012). However, all pro-angiogenic therapeutic strategies reaching clinical trials have had disappointing results (Chu and Wang, 2012). While the reason for failure is specific for each therapeutic approach, many can be attributed to the classic challenges of drug delivery: failure to deliver the therapeutic to the target tissue at the necessary doses and for the required duration, while avoiding degradation and delivery to off-target tissues (Bader and Putnam, 2014).

### Protein therapeutics

Pro-angiogenic approaches have largely focused on delivery of angiogenic proteins including but not limited to vascular endothelial-, fibroblast-, or platelet-derived growth factor (Losordo and Dimmeler, 2004; Papanas and Maltezos, 2007). Delivery of these factors is considered one of the more straightforward pro-angiogenic approaches; it is simpler and more controllable than cell- and gene-based therapies, and many pro-angiogenic proteins are commercially available (Chu and Wang, 2012). However, simple injection is an inefficient and ineffectual delivery method, as proteins suffer from poor localization and rapid clearance (Laham et al., 1999). These drawbacks present significant challenges, as tight spatio-temporal control over pro-angiogenic proteins, such as VEGF, is required to induce formation of stable and functional vessels (Ozawa et al., 2004; Silva and Mooney, 2007). Additionally, as angiogenesis is a highly regulated process controlled by a number of growth factors, some work suggests that delivery of multiple pro-angiogenic proteins that more closely recapitulate the pro-angiogenic signaling cascade may be required to produce therapeutically relevant and long-lasting vascularization (Mooney et al., 2007; Sylven et al., 2007; Layman et al., 2009; Brudno et al., 2013). While many recombinant human proteins do not elicit a notable immune response in clinical trials, some have induced an immune reaction substantial enough to prevent their use, underlying the importance of addressing this possibility in translational studies (Porter, 2001).

### Peptide therapeutics

As peptides have smaller sequences than proteins (generally <50 amino acids), peptides can be produced either synthetically or grown biologically in *Escherichia coli* or yeast, giving them more versatile production schemes than proteins (Lehninger et al., 2000). Their smaller size allows peptides to be delivered at higher concentrations to target tissue. Additionally, peptides often do not require complex tertiary structures for bioactivity (Finetti et al., 2012). While some pro-angiogenic peptides consist of entirely novel sequences (Hardy et al., 2008), many mimic the bioactive region of pro-angiogenic growth factors (Lane et al., 1994; Finetti et al., 2012) or the extracellular matrix (Demidova-Rice et al., 2011, 2012), facilitating rationally designed therapeutic sequences. There are many modifications to peptides that can be made to increase their thermal and protease stability, such as cyclization, substitution of amino acids not critical for biological effects, and use of non-natural amino acids (Rozek et al., 2003; Diana et al., 2008; Gentilucci et al., 2010). Peptide sequences have been identified that are sensitive to protease cleavage (West and Hubbell, 1999; Patterson and Hubbell, 2010), and that enhance cell penetration and uptake (Lindgren et al., 2000; Copolovici et al., 2014), which are attractive for use in drug delivery applications. Together, these many advantages make peptides an attractive drug class for any number of therapeutic applications.

However, there are drawbacks to the use of peptide drugs. In some situations, peptides do not fully retain the bioactivity of the parent protein and must be delivered at higher doses than protein counterparts to achieve similar effects (Ben-Sasson et al., 2003). This is not always the case, and some peptides afford comparable bioactivities to the parent protein (Santulli et al., 2009). Peptides are still susceptible to protease degradation (Frackenpohl et al., 2001), and similar to proteins, peptides suffer from rapid clearance by the liver and kidneys, leading to poor pharmacokinetics when delivered systemically (Vlieghe et al., 2010; Craik et al., 2013). Peptides that act intracellularly may have difficulty penetrating the hydrophobic cell membrane, reducing their efficacy (Copolovici et al., 2014). Similar to proteins, peptides may elicit an immune response (Niman et al., 1983), and flexible peptide conformations can result in off-target receptor interactions (Vlieghe et al., 2010). These drawbacks have likely contributed to the delayed development and approval of peptides as compared to small molecule and antibody-based therapeutics (Kaspar and Reichert, 2013). However, new synthetic strategies, increased interest in drugs delivered via routes beyond oral and parenteral routes, and the development of improved delivery systems have recently increased their popularity (Vlieghe et al., 2010).

This renewed interest in therapeutic peptides has resulted in the identification and use of peptides as pro-angiogenic therapies, as well as a number of other applications. In 2011, over 500 peptides were in pre-clinical studies, and as of 2013, there were 128 therapeutic peptides in the FDA-approval pipeline: 40 in phase I, 74 in Phase I/II or Phase II, and 14 in Phase II/III or Phase III trials. The peptides currently in clinical trials are designed to treat a variety of diseases, including cancers, acute bacterial infections, type 2 diabetes, osteoporosis, and chronic foot ulcers (Kaspar and Reichert, 2013; Thomas et al., 2014). The number of therapeutic peptides that have been identified but are still in pre-clinical trials is even greater, and they too encompass a variety of therapeutic actions, including chemotherapeutic (Selivanova et al., 1997; Yang et al., 2003) and anti-inflammatory (Akeson et al., 1996; Schultz et al., 2005) peptides, as well as the pro-angiogenic peptides, which are of primary interest here (Lane et al., 1994; Demidova-Rice et al., 2012; Finetti et al., 2012). Select therapeutic peptides, their sources, and current phases of development are listed in Table 1, and a number of pro-angiogenic peptides that have shown promising results are summarized in Table 2, with specific interesting examples further discussed here.

**Table 1:** **Examples of therapeutic peptides**.

Therapeutic application	Name	Source	Phase of development	Reference
Wound healing	DSC127	Angiotensin (1–7)	Phase III	Rodgers and Dizerega (2013), Derma Sciences (2015)
	GHK	Cu^2+^ binding region of SPARC	Failed phase III trials for venous stasis ulcers	Pickart (2008)
Cosmetic	GHK (and analogues)	Cu^2+^ binding region of SPARC	FDA-approved for both wrinkle treatment and hair regrowth	Pickart (2008)
Anti-inflammatory	AF12198	Phage display	Pre-clinical	Akeson et al. (1996), Mandrup-Poulsen (2012)
	CBX129801	Cleavage product of proinsulin	Phase IIb	Henriksson et al. (2005), Cebix (2013)
Chemotherapeutic	Endostatin peptide fragment I (180–199)	Collagen XVIII	Pre-clinical	Olsson et al. (2004)
	VEGF-derived peptide	Exon 6a of VEGF gene	Pre-clinical	Lee et al. (2010)
	ATN-161	Fibronectin	Phase II	Plunkett et al. (2002), Cianfrocca et al. (2006)
Osteoporosis	BA058	Parathyroid hormone receptor (PTHR) agonist	Phase III	Radius Health (2015)
Anti-bacterial	Oritavancin (LY333328)	Semisynthetic lipoglycopeptide analogue of vancomycin	FDA approved	Zhanel et al. (2012), FDA (2014)

*A selection of bioactive peptides and intended therapeutic applications*.

**Table 2:** **Pro-angiogenic peptides**.

Pro-angiogenic peptide	Sequence	Source	Demonstrated effects	Reference
Qk	KLTWQELYQLKYKGI	α-helix region of VEGF	Causes similar signaling and *in vitro* effects to full-length VEGF. *In vivo*, Qk increased vessel density in ischemic hind limbs and Matrigel plugs, as well as the rate of cutaneous wound closure	Santulli et al. (2009), Finetti et al. (2012)
PAB2-1c	(C*VRKIEIVRKK)_2_–Ahx–Ahx–Ahx–RKRKLERIAR–NH_2_	Mimic of PDGF	Stimulates cell proliferation, migration, and collagen gel contraction similar to full-length PDGF *in vitro*	Lin et al. (2007)
T7 vasculotide	(PEG-CHHHRHSF) tetramer	Tie-2-binding region of Ang1	Increases serum-free cell survival and cell migration as compared to controls *in vitro*. Increases vessel number and size when delivered from Matrigel, and increase the rate of diabetic wound closure when delivered using Intrasite Topical Gel *in vivo*	Van Slyke et al. (2009), Slyke (2011)
GHK, GHK-Cu, or SPARC_120-122_	GHK	Cu^2+^-binding region of SPARC	Induces a wide range of cellular effects, including reducing inflammatory while increasing anti-inflammatory factors, increasing extracellular matrix protein production, and matrix metalloproteinase expression. *In vivo* effects have been shown ranging from increasing vascularization in the rabbit eye, increasing the rate of uncomplicated and diabetic wound healing, and inhibiting gastric ulcer formation	Pickart (2008)
Comb1	DINECEIGAPAGEETEVTVEGLEPG	Combination of the epidermal growth factor -like domains of fibrillin 1 and tenascin X	Increases cell proliferation, tube formation, and sprouting compared to controls *in vitro*. Increased chemically impaired cutaneous wound healing when co-delivered daily with UN3	Demidova-Rice et al. (2011, 2012)
UN3	NH_2_-ELLESYIDGRPTATSEYQTFFNPR-amide	Previously unknown peptide fragment from platelet lysate	Significantly increased cell migration, proliferation, and tube formation *in vitro*. Significantly increased vessel density in impaired cutaneous wounds. Increased chemically impaired cutaneous wound healing when co-delivered daily with Comb1	Demidova-Rice et al. (2012)
KRX-725	MRPYDANKR	Second intercellular loop of sphingosine 1-phosphate (S1P) 3	Increases aortic ring sprouting as compared to controls with greater smooth muscle cell co-localization to endothelial cells than VEGF. Increases in vascularization of the rabbit cornea were obtained by co-treatment with KRX-725 and VEGF or bFGF as compared to factors alone	Ben-Sasson et al. (2003)
Pep-12	NYLTHRQ	Ig-like domain II of VEGF receptor 1	Facilitates integrin-mediated cell adhesion and cause tube formation *in vitro*. Significantly increases angiogenesis in the rabbit cornea as compared to controls, albeit to a lesser extent than VEGF	Soro et al. (2008)
LL-37	LLGDFFRKSKEKIGKEFKRIVQRIKDFLRNLVPRTES	The 134–170 amino acid region of the human cationic anti-microbial protein 18	Originally identified as an anti-microbial peptide produced in response to inflammation or infection, it was shown to have pro-angiogenic effects in addition to anti-microbial action. LL-37 caused dose-dependent increases in cell proliferation and increased collateral blood flow, capillary density, and blood velocity in a rabbit hind-limb ischemia model	Koczulla et al. (2003)
YR or RoY	YPHIDSLGHWRR	Identified by phage display for binding to endothelial cells	Increased cell proliferation and migration *in vitro*. Increases vessel density when injected into a mouse ear and hindlimb reperfusion when delivered intramuscularly	Hardy et al. (2007, 2008)
AcSDKP	AcSDKP	A naturally expressed regulator of hematopoiesis found in bone marrow	AcSDKP increases cell migration and tube formation, with increasing then decreasing responses as the concentration is increased beyond the optimal dose. Similar results were seen *in vivo* using the Matrigel plug assay, with greater vascularization induced with 10^−9^ M than 10^−5^ M of peptide	Liu et al. (2003)

A selection of pro-angiogenic peptides, all which are in pre-clinical testing. Standard amino acid abbreviations are used. C*, disulfide bridge; Ahx, aminohexanoic acid; Ac, acetyl.

#### Qk

The potent pro-angiogenic peptide Qk was designed to mimic the receptor binding α-helix region of VEGF, a key factor in the early stages of angiogenesis (Figure 1). Based on the 17–25 amino acid region of VEGF, Qk was strategically modified such that it would maintain the α-helix secondary structure of the corresponding segment of the full-length protein and the three-dimensional presentation of amino acids critical for VEGF receptor interactions. Qk is able to induce ERK1/2 and Akt phosphorylation similar to full-length VEGF, and results in similar cell proliferation and migration *in vitro* (D’Andrea et al., 2005; Diana et al., 2008; Finetti et al., 2012). While more stable than VEGF_17–25_, Qk still has a serum half-life of only ~4 h, making simple injection an inefficient method to maintain therapeutic levels of bioactive peptide (Finetti et al., 2012). As spatial and temporal control over VEGF concentration is critical for vessel formation (Mooney et al., 2007), it is reasonable that Qk would require similar control. This need for controlled delivery of this peptide is emphasized by the controlled release systems exploited in the *in vivo* studies performed with Qk. An infusion pump was used to deliver Qk to ischemic hind limbs, increasing vessel density; Matrigel and Pluronic gels have been used to sustain the delivery of Qk subcutaneously and to cutaneous wounds, increasing vessel density and the rate of wound closure, respectively (Santulli et al., 2009).

#### PAB2-1c

PAB2-1c was designed to mimic PDGF, a protein involved in vessel detachment and sprouting, pericyte recruitment, and vessel maturation and remodeling (Figure 1) (Lin et al., 2007). PAB2-1c was shown to bind PDGF receptors α and β and induce Akt and ERK1/2 phosphorylation, albeit to a lesser extent than full-length PDGF. Additionally, while PAB2-1c induced similar cell proliferation and migration, higher doses of the peptide were required to match the full-length protein (Lin et al., 2007). While no *in vivo* data have been published exploiting this peptide, it holds great potential for future applications attempting to more mimic the pro-angiogenic signaling cascade shown in Figure 1. For example, materials could be developed by delivering peptides that first stimulate the early phases of vessel development (i.e., the VEGF mimic Qk) followed by peptides that stimulate pericyte recruitment and vessel remodeling (i.e., the PDGF mimic PAB2-1c). Additionally, PAB2-1c is a multi-domain peptide, containing two copies of PDGF-BB_153–162_ as well as a heparin-binding domain RKRKLERIAR (Verrecchio et al., 2000), which could be exploited for controlled release purposes (further discussed below).

#### GHK

The secreted protein acidic and rich in cysteine (SPARC) (also known as osteonectin) is an extracellular matrix protein expressed during embryogenesis and tissue repair/remodeling. *In vivo*, SPARC is cleaved by proteases into distinct fragments, with fragments from each domain producing drastically different cellular responses (Motamed, 1999). Fragments from the cysteine-rich follistatin-like region that contain the copper-binding sequence GHK have been shown to have numerous pro-angiogenic and healing effects, increasing fibroblast production of VEGF and FGF, increasing extracellular matrix production and remodeling, increasing vessel formation in the rabbit cornea, accelerating dermal wound healing, increasing hair follicle growth, and acting as a chemoattractant for macrophages, capillary cells, and mast cells, to name a few (Pickart, 2008). While the delivery system, dose, and model varies widely across the many studies exploiting GHK and its analogues, the peptide is generally delivered using a controlled release system or by repeated administration (Pickart, 2008). Interestingly, when coupled to alginate hydrogels, GHK increased VEGF and FGF production by mesenchymal stem cells (Jose et al., 2014), potentially increasing their pro-angiogenic efficacy and capacity for tissue repair (Rustad et al., 2012; Hoffman et al., 2013). Together, the numerous and diverse effects of this peptide make it an intriguing drug for use in pro-angiogenic, wound repair, and tissue engineering applications.

#### Synergistic Effects Upon Delivery of Multiple Factors

Numerous peptides have improved efficacy upon co-delivery with other peptides or factors. Qk caused synergistic increases in cell migration when delivered with VEGF or FGF-2 (Finetti et al., 2012). While UN3 (a peptide fragment identified from platelet lysate) alone was able to increase vascularization of cutaneous wounds, increased wound quality was only observed when UN3 was co-delivered with Comb1 (a combination of the fibrillin 1 and tenascin X) (Demidova-Rice et al., 2012). KRX-725 showed similar additive effects when co-delivered with bFGF, significantly increasing vascularization of the rabbit cornea as compared to delivery of the peptide or protein alone (Ben-Sasson et al., 2003). This suggests that pro-angiogenic peptides, similar to their protein counterparts, could benefit from controlled release strategies that deliver multiple factors, either all peptides, or a combination of peptides and proteins.

Delivery of multiple proteins from a material that more closely replicating their temporal expression in the pro-angiogenic signaling cascade has been shown to improve pro-angiogenic effects as compared to singular protein delivery, or delivery of multiple proteins without this temporal control (Mooney et al., 2007; Brudno et al., 2013). For example, delivery of VEGF followed by PDGF, which more closely recapitulates the native pro-angiogenic healing cascade (Figure 1), improves vessel density, size, and maturity as compared to delivery of either factor alone (Richardson et al., 2001; Sylven et al., 2007). Similarly, delivery of multiple pro-angiogenic (VEGF and Ang2) followed by pro-maturation (PDGF and Ang1) factors with temporal delivery motivated by healthy angiogenic signaling-induced formation of more mature, larger vessels than controls (Brudno et al., 2013). As previously discussed, one could envision exploiting peptide mimics of these two factors (Qk and PAB2-1c) similarly. Additionally, the Ang1 mimic T7 could be employed as a pro-maturation peptide delivered in conjunction with a pro-angiogenic peptide, such as Qk, as Ang1 is important for pericyte recruitment and vessel maturation/remodeling (Figure 1).

## Drug Delivery Systems for Pro-Angiogenic Peptides

Drug delivery systems are often used to address delivery challenges associated with therapeutic efficacy. While the specific goal of each delivery system depends on the drug being delivered and its target tissue/disease state, the over-arching goal is to maintain or increase the efficacy of the therapeutic while minimizing or eliminating toxicity and side effects (Bader and Putnam, 2014). To achieve these goals, drug delivery systems can be designed to improve the solubility of the drug, protect it from degradation, increase its circulation/retention time, improve preferential tissue accumulation, and/or prolong its retention at the target site (Bader and Putnam, 2014). Of particular concern when delivering pro-angiogenic drugs is off-target delivery, which could potentially encourage the development of tumors (Carmeliet and Jain, 2000), or increasing the severity of diseases associated with excessive angiogenesis, such as macular degeneration (Kent, 2014).

The most commonly exploited drug delivery systems can be broadly classified into three categories: orally delivered, soluble (or injectable), and depot-based implantable systems. Delivery of proteins and peptides using alternate entry routes (e.g., nasal, pulmonary, and transdermal) are not commonly exploited for pro-angiogenic applications, and are reviewed elsewhere (Agu et al., 2001; Shoyele and Cawthorne, 2006; Antosova et al., 2009). Methods to improve oral delivery of protein and peptide drugs have been recently reviewed (Al-Hilal et al., 2013; Renukuntla et al., 2013). Du and Stenzel have published a thorough review that focuses on chemical conjugation methods for peptide drug delivery using soluble polymeric delivery systems (liposomes, nanoparticles, etc.) (Du and Stenzel, 2014). These soluble and oral delivery systems are less desirable for pro-angiogenic applications, such as cardiac ischemia and diabetic wounds, due to difficulty achieving preferential accumulation at target tissues, and previously mentioned concerns over systemic delivery of pro-angiogenic factors encouraging tumor development (Carmeliet and Jain, 2000).

### Depot-based drug delivery systems

Depot-based delivery systems are associated with improved patient compliance and have been successfully used for decades for longitudinal delivery of drugs, such as contraceptives (Graesslin and Korver, 2008). This delivery route avoids the need for the drug to pass through the harsh conditions of the digestive system, and through the intestinal epithelium. Placing the drug delivery depot directly at the target tissue site allows preferential delivery to the target tissue, achieving higher doses and reducing unwanted off-target tissue side effects. Additionally, by designing the depot to contain a high dose of drug and slowly release it over time, a single treatment can maintain drug dose within the therapeutic window for extended durations (Bader and Putnam, 2014).

Osmotic pumps are commonly used to achieve prolonged drug delivery (Santulli et al., 2009; Bader and Putnam, 2014). However, pumps must be removed after payload delivery, necessitating additional surgeries. Biomaterial-based peptide delivery systems that provide longitudinal release are an alternate method to locally deliver proteins and peptides, and the use of degradable biomaterials avoids the need for surgical recovery. While many depot-based delivery systems are formed externally and then implanted, some biomaterials allow for *in situ* formation, where precursor material can be injected and then polymerized in place, allowing for minimally invasive implantation (Anseth et al., 2002), particularly attractive for cardiac applications.

**Figure 2:**
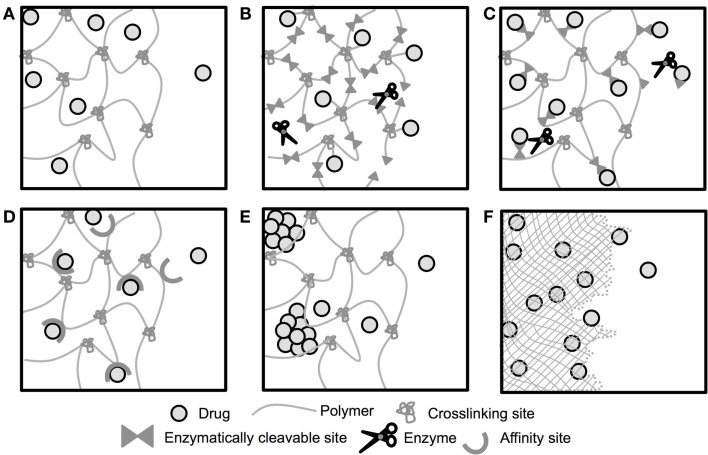
**Schematic of drug release from biomaterial depots**. Release of drugs from depot-based biomaterials can be controlled by a number of mechanisms. **(A)** Drug is encapsulated within a biomaterial with large enough mesh/pore size to allow for diffusive release of the encapsulated drug. **(B)** Drug is tethered to a biomaterial that degrades in response to enzyme expression and releases the drug upon degradation of the biomaterial. **(C)** Drug is tethered to the biomaterial by the enzymatically cleavable tether, and released upon linker cleavage. **(D)** Diffusive release of encapsulated drug is prolonged by affinity interactions between the material and the drug. **(E)** Diffusive release of encapsulated drug is prolonged by delayed dissolution of the drug. **(F)** Drug is encapsulated within a degradable biomaterial and released as the material degrades. Not to scale.

Depot-based methods present many specific advantages for pro-angiogenic therapies, including providing localized drug delivery thereby reducing concerns over off-target effects (Chu and Wang, 2012). Depot methods can also provide the spatial and temporal delivery of factors necessary for the development of stable, functional vessels (Mooney et al., 2007; Brudno et al., 2013). A schematic depicting select modes drugs have been released from biomaterial depots is shown in Figure 2. To date, the pro-angiogenic biomaterials field has largely focused on delivery of full-length proteins, and few depot-based methods for peptides have been developed (Du and Stenzel, 2014). This is likely due to the previously discussed historical difficulties associated with the use of peptide drugs. However, the recent identification of numerous pro-angiogenic peptides (Table 2) combined with new and improved peptide synthesis strategies have opened up an entirely new class of pro-angiogenic drugs for delivery. Herein, we focus on those biomaterials that have been developed for delivery of pro-angiogenic peptides, and those that could be easily adapted for peptide delivery, laying the foundation for a whole host of potential pro-angiogenic therapies.

#### Hydrogels

Hydrogels are highly hydrated crosslinked polymeric networks often used to provide sustained, localized drug delivery. The highly hydrated nature of hydrogels is similar to native tissues, and the aqueous network can stabilize peptide and protein drugs. Depending on the polymer used to form the gel, hydrogels can have highly tunable physical and chemical properties, to provide a wide degree of control over hydrogel properties and drug release behavior (Lin and Anseth, 2009b; Liechty et al., 2010).

Hydrogels can be formed using a variety of approaches. These include physical crosslinks (entanglements, hydrogen bonding, or hydrophobic forces), covalent bonds, ionic crosslinks, or a combination of these approaches (Peppas et al., 2006). These gels may be physically stable, or may degrade or dissolve, either due to the nature of the polymer used, or as a result of specific chemical functionalities introduced for degradability. While a wide range of polymers have been used to form hydrogels, they can be broadly classified as either natural or synthetic polymers, although “hybrid hydrogels” can be formed that use both natural and synthetic polymers (Slaughter et al., 2009). Select examples of hydrogels used for controlled drug delivery are listed in Table 3.

**Table 3:** **Hydrogel-based biomaterials for controlled drug delivery**.

Type	Polymer	Drug delivered	Drug type	Mode of release	Reference
Natural hydrogels	Alginate	VEGF	Protein	Diffusive	Silva and Mooney (2007, 2010)
	Alginate	VEGF and PDGF	Dual proteins	Diffusive	Sylven et al. (2007)
	Fibrin	VEGF	Protein	Proteolytic degradation	Ehrbar et al. (2004)
	Extracellular matrix	bFGF	Protein	sGAG-binding affinity	Seif-Naraghi et al. (2012)
	Extracellular matrix	HGF-f	Protein fragment	sGAG-binding affinity	Sonnenberg et al. (2015)
	Gelatin	FGF-2 and G-CSF	Dual proteins	Diffusive and ionic interactions	Layman et al. (2009)
	Hyaluronic acid	TGF-β1	Protein	Heparin-binding affinity	Jha et al. (2015)
	Matrigel	T7 Vasculotide	PEG-peptide tetramer	Diffusive	Van Slyke et al. (2009)
	Matrigel	Qk	Peptide	Diffusive	Santulli et al. (2009)
Synthetic hydrogels	Poly(ethylene glycol) multiacrylate and dithiolthreitol	hGH	Protein	Dissolution and diffusion	van de Wetering et al. (2005)
	Poly(ethylene glycol vinyl sulfone and proteolytically cleavable peptide	VEGF	Protein	Enzymatically responsive	Zisch et al. (2003)
	Poly(ethylene glycol) diacrylate and proteolytically cleavable peptide	VEGF	Protein	Enzymatically responsive	Phelps et al. (2010)
	Poly(ethylene glycol) norbornene and enzymatically cleavable peptide	Qk, SPARC_113_, SPARC_118_, and model peptides	Peptide	Enzymatically responsive	Van Hove et al. (2014)
	Poly(*N*-isopropylacrylamide-co-propylacrylic acid-co-butyl acrylate)	bFGF	Protein	pH-responsive and diffusive	Garbern et al. (2010), Murry et al. (2011)
Combinatory/hybrid hydrogels	Poly(ethylene glycol)-bis-butanoic acid and hydrazide-functionalized heparin	VEGF	Protein	Heparin-binding affinity	Tae et al. (2006)
	Multi-arm poly(ethylene glycol) thiol and dextran vinyl sulfone	IgG, BSA, Lysozyme, and bFGF	Protein	Diffusive	Hiemstra et al. (2007)
	Poly(ether)urethane–polydimethylsiloxane + fibrin	VEGF and bFGF	Dual proteins	Diffusive	Losi et al. (2010)
	Hyaluronic acid + PEG	VEGF and bFGF	Protein	Heparin-binding affinity	Pike et al. (2006)

*Select examples of hydrogel-based biomaterials used for controlled drug delivery*.

Generally speaking, the rate of drug release from hydrogels is controlled by the diffusion of drug out of the crosslinked gel network (Figure 2A) (Slaughter et al., 2009). However, diffusion alone often does not facilitate long-term delivery of small drugs, such as peptides and small molecule drugs, as hydrodynamic radius is proportional to release rate, resulting in faster release relative to larger molecules, such as proteins (Lustig and Peppas, 1988). Therefore, to deliver these small drugs from hydrogels, more advanced modifications are often required to control the release (Figures 2B–E).

##### Naturally derived polymers

Polymers from natural sources, such as alginate, agarose, chitosan, collagen, digested extracellular matrix, fibrin, gelatin, and hyaluronic acid (HA) can be used to form hydrogels. As they are derived from plant or animal sources, natural polymers generally have low toxicity and good biocompatibility. However, the physical and chemical properties of naturally derived hydrogels can be difficult to control. Additionally, due to their biological sources, these materials often present signals that can be recognized by cells within the body, which can be advantageous or disadvantageous depending upon the application (Peppas et al., 2006).

###### Alginate

Alginate is a naturally occurring linear polysaccharide that is soluble in water, but due to negatively charged side groups can be ionically crosslinked by the addition of divalent cations, such as Ca^2+^. While they are generally biocompatible, without additional modification alginate hydrogels undergo slow and uncontrolled degradation *in vivo* (Bouhadir et al., 2001; Silva and Mooney, 2007). Alginate hydrogels have been used for the controlled delivery of growth factors in a number of studies, and have shown the importance of extended delivery of VEGF to ischemic tissue (Silva and Mooney, 2007, 2010). Additionally, alginate hydrogels delivering VEGF followed by PDGF resulted in the same capillary density within infarcted tissues versus gels delivering VEGF, but significantly improved the number of mature vessels over gels delivering either PDGF or VEGF alone (Sylven et al., 2007), demonstrating the improvement in angiogenesis that can be obtained by more closely recapitulating the natural pro-angiogenic signaling cascade reviewed in Figure 1. While able to controllably deliver large proteins, unmodified alginate hydrogels are not well suited for delivery of peptide drugs. The mesh size of alginate hydrogels varies depending on the percentage of alginate used, but is generally on the order of magnitude of 10 nm (Turco et al., 2011). This facilitates hindered diffusion of larger proteins, such as VEGF and PDGF, but would likely be less successful delivering small peptides.

###### Extracellular matrix

Hydrogels derived from decellularized, digested extracellular matrix (ECM) have also been exploited for delivery of pro-angiogenic factors. These materials provide a physical structure that supports cell infiltration and vascularization and provides structural support to the tissue, which has been shown beneficial in limiting post-myocardial infarction damage to cardiac tissue (Okada et al., 2010; Singelyn et al., 2012). These materials are highly heterogeneous, and some ECM-based materials have caused inflammatory responses *in vivo* (Seif-Naraghi et al., 2012), while others reduce the extent of chronic inflammation (Faulk et al., 2014). Decellularized, digested porcine pericardiac tissue has been exploited for the sustained delivery of bFGF. Release of bFGF from the ECM material occurred at approximately half the rate as from collagen gels, likely due to affinity interactions between bFGF and sulfated glycosaminoglycans (GAGs) within the ECM (Figure 2D). Upon injection into cardiac tissue, approximately three times more bFGF was retained after 5 days when the protein was delivered in the ECM material compared to direct injection. Additionally, the bFGF-releasing ECM significantly increased the number of small (10–50 μm) vessels within the tissue. However, the bFGF-releasing ECM caused a significant increase in inflammation, undesirable in many tissue repair applications (Seif-Naraghi et al., 2012).

Similar porcine pericardial ECM hydrogels were used for sustained delivery of a hepatocyte growth factor (HGF) fragment (HGF-f), which shows similar bioactivity to full-length HGF while being less than half the size of the full-length protein (Liu et al., 2014). The ECM-based hydrogel provided sustained release of HGF-f, releasing ~30% over 5 days and significantly increased arteriole density in infarcted cardiac tissue. However, it only caused trending improvements in function as assessed by ejection fraction (Sonnenberg et al., 2015). The HGF-f released from these gels is still substantial larger than a peptide (~40 kDa), and sustained delivery of smaller peptides from these hydrogels would likely not occur without similarly exploiting GAG affinity interactions. This would require modification of the peptide with a heparin-binding sequence, such as RKRKLERIAR (Lin et al., 2007), (XBBXBX)_n_, or (XBBXXBX)_n_, where X is uncharged or hydrophobic, and B is a basic amino acid (Verrecchio et al., 2000). Additionally, any inflammatory reaction to these ECM materials would need to be addressed, as this is a significant concern for translation.

###### Fibrin

Fibrin hydrogels have been used for a number of biomaterial and drug delivery applications. Similar to fibrin clots formed after vascular injury, fibrin hydrogels are formed by reacting fibrinogen and thrombin (Ehrbar et al., 2004; Schmoekel et al., 2005). They are highly biocompatible, and can be degraded by plasmin and other enzymes in the body (Ye et al., 2000; Ahmed et al., 2007). In an attempt to improve the efficacy of VEGF by providing long-term delivery of the growth factor, Ehrbar et al. conjugated VEGF to a fibrin matrix such that the VEGF molecule could only be released when the fibrin matrix was proteolytically degraded (Figure 2B). This greatly extended the duration of VEGF release, and the fibrin-released VEGF increased the formation of new arterial and venous structures within the chick chorioallantoic membrane, while passively released VEGF (Figure 2A) primarily resulted in chaotic changes to the vasculature (Ehrbar et al., 2004). This enzymatically responsive gel could easily be adapted for delivery of pro-angiogenic peptides. By including the factor XIIIa substrate NQEQVSPL onto either the C- or N-termini of the peptide, peptides could similarly be covalently integrated into the fibrin network via factor XIIIa activation (Zisch et al., 2001). However, proteins/peptides released from these gels contain residual fragments of the fibrin gel, which could affect bioactivity. Testing of released VEGF showed comparable bioactivity to non-tethered, encapsulated protein (Ehrbar et al., 2004), but this would not necessarily be the case for all drugs.

One disadvantage to the use of fibrin gels is that they do not afford control over the rate of gel degradation and associated drug release. Some degree of control over the time course of VEGF release was achieved by introducing a plasmin-sensitive substrate between the growth factor and the Factor XIIIa substrate (NQEQVSPL-LIK↓MKP-VEGF, ↓ indicates cleavage site) (Ehrbar et al., 2005). However, this modification to the system only accelerated growth factor release by ~25%, and does not provide a means to easily tune protein/peptide release kinetics.

###### Gelatin

Gelatin is a natural hydrogel derived from collagen used in drug delivery applications because of its biocompatibility and controllable degradation (Tabata and Ikada, 1998; Young et al., 2005). Covalently crosslinked gelatin hydrogels were exploited for the controlled delivery of FGF-2 and granulocyte-colony stimulating factor (G-CSF). While release of both FGF-2 and G-CSF were diffusion mediated, FGF-2 release was delayed as compared to G-CSF, likely due to ionic interactions between the anionic gelatin and cationic FGF-2 (Figures 2A,D). Hydrogels releasing both growth factors improved ischemic hind limb reperfusion assessed via increased capillary density and maturity as compared to PBS or singularly delivered growth factor controls (Layman et al., 2009). By processing collagen in either acidic or alkaline conditions, its isoelectric point can be modified. This allows oppositely charged molecules to interact with the gelatin and form a polyion complex, extending release by affinity interactions (Tabata and Ikada, 1998; Young et al., 2005). Yamamoto et al. showed this when they demonstrated that encapsulation of bFGF and transforming growth factor- β1 (TGF-β1) in acidic gelatin hydrogels prolonged *in vivo* delivery as compared to direct injection. However, despite similar isoelectric points, prolonged delivery of bone morphogenetic protein-2 (BMP-2) and VEGF was not achieved possibly due to differences in 3D structure and charge exposure (Yamamoto et al., 2001). In addition to being unable to deliver the potent pro-angiogenic protein VEGF, these gelatin hydrogels are not ideal for the delivery of smaller peptides. The strength of the interaction between the drug and the gelatin decreases as the size of and number of charges on the drug being delivered decreases (Tabata and Ikada, 1998), making it likely that gelatin would not prolong the delivery of smaller peptides. This was illustrated by Saramento et al., who attempted to use polyion interactions to deliver insulin, which at 51 amino acids is on the cusp of what is considered a peptide versus a protein (Sarmento et al., 2007). By combining this negatively charged peptide with negatively charged fibrin and positively charged chitosan, nanoparticles were formed crosslinked by electrostatic forces. However, release of the peptide was rapid, with ~60% released in 2 h, demonstrating the limitations of using ionic interactions to control the release of small peptides (Sarmento et al., 2007).

###### Hyaluronic acid

Hyaluronan or HA is a naturally occurring component of the extracellular matrix. HA degradation *in vivo* is mediated by hyaluronidases, six enzymes that hydrolyze HA (Stern, 2004). The released HA fragments have been shown to have pro-angiogenic effects *in vivo* (Montesano et al., 1996), making it a promising material for delivery of pro-angiogenic factors, as both the material and drug being delivered could contribute to the desired pro-angiogenic response. However, this convolutes drug-specific effects, and excessive pro-angiogenic signaling can sometimes lead to the development of leaky vasculature (Yancopoulos et al., 2000). HA hydrogels with mesh sizes and degradation controllable based upon the degree of HA methacrylation can be formed by UV polymerization. These hydrogels exhibit good biocompatibility upon subcutaneous implantation (Leach et al., 2003) but their mesh sizes are very large (~600 nm), causing rapid release of encapsulated proteins (Leach and Schmidt, 2005) and making this an unattractive approach for delivery of small pro-angiogenic peptides. Enzymatically degradable HA hydrogels were formed by functionalizing HA with acrylate groups and reacting with di-thiol containing matrix metalloproteinase (MMP)-degradable crosslinking peptides and thiolated heparin for affinity-controlled protein release (Figure 2D). Release of encapsulated TGF-β1 was prolonged over >3 weeks, with the release rate affected by the molecular weight of heparin used, as well as the amounts of heparin and TGF-β1 used in hydrogel formation (Jha et al., 2015). While this study did not investigate *in vivo* degradation or the pro-angiogenic/wound healing effects of the material, it did demonstrate the highly tunable protein release that can be achieved using heparin-functionalized HA hydrogels. However, due to the number of growth factors that have affinity for heparin (Peysselon and Ricard-Blum, 2014), it is possible that host proteins with greater affinity for heparin may displace drug molecules when introduced *in vivo*.

Supramolecular interactions of adamantine and cyclodextrin have also been exploited to form shear-thinning, self-healing HA hydrogels, by combining adamantane- and cyclodextrin-functionalized HA. The physical properties of these HA gels, such as stiffness and degradation rate, can be modified by varying the weight percentage of HA, as well as the extent of functionalization (Rodell et al., 2013). These hydrogels can be rendered enzymatically degradable by tethering the adamantane to the HA by an MMP-degradable peptide sequence (Rodell et al., 2015). This material holds great promise for the delivery of pro-angiogenic peptides due to the affinity interactions of cyclodextrin with peptides (Tiwari et al., 2010), particularly those containing hydrophobic and aromatic amino acids (Castronuovo et al., 1995; Aachmann et al., 2012). However, due to these fairly non-specific interactions, these materials have the same potential concern as heparin gels whereby host molecules may displace drug molecules, drastically affecting drug release. Many HA hydrogels are formed using poly(ethylene glycol) (PEG) crosslinkers, and are further discussed in the Section “Hybrid materials” below.

###### Matrigel

Matrigel is a mixture of extracellular matrix molecules produced by Engelbreth–Holm–Swarm (EHS) mouse sarcoma cells that is soluble at 4°C but polymerizes when incubated at 37°C. Due to the gentle polymerization conditions required to form gels, and ability of a variety of cells to interact with the gel during vascularization, Matrigel is commonly used *in vivo* to provide diffusive release (Figure 2A) and evaluate the efficacy of pro- and anti-angiogenic proteins and peptides (Kleinman and Martin, 2005; Santulli et al., 2009; Van Slyke et al., 2009). However, Matrigel has inherent biological activity that varies between production lots based on residual-growth factors left in the matrix (Kleinman and Martin, 2005). Concerns over its tumor source prevents Matrigel from being used for translational drug delivery purposes, and many studies using Matrigel to deliver therapeutic factors *in vivo* do not characterize the release of the factor from the gel (Santulli et al., 2009; Van Slyke et al., 2009).

##### Synthetic polymers

Many synthetic polymers have also been exploited for controlled drug delivery. As compared to natural polymers, synthetic polymers afford a greater degree of control over resulting hydrogel networks. Hydrogel properties, such as crosslinking density, mechanical strength, degradation, drug release profile, and even stimuli-responsive behavior can be controlled by altering the composition of the polymer network. While many synthetic polymers are bio-inert, they are frequently engineered to incorporate functional groups that allow cells to bind to and interact with the hydrogel (Peppas et al., 2006).

###### Poly(ethylene glycol)

Poly(ethylene glycol) hydrogels are a commonly used synthetic biomaterial for drug delivery (Peppas et al., 2006; Lin and Anseth, 2009b; Slaughter et al., 2009). PEG hydrogels are highly hydrophilic, inert, and biocompatible, and PEG has been approved by the FDA for a number of clinical uses (Peppas et al., 2006). Additionally, PEG hydrogels have been shown to have highly tunable degradation profiles and mechanical properties (Lin and Anseth, 2009b). While PEG hydrogels are inherently bio-inert, they can be functionalized with cell adhesion molecules, such as the RGD peptide to facilitate cellular interactions (Hern and Hubbell, 1998).

To facilitate hydrogel formation, PEG can be crosslinked by two mechanisms: step-growth and chain-growth, or a combination of the two, termed mixed-mode. Chain-growth polymerization occurs when PEG macromers contain self-reactive terminal groups (predominantly acrylates and methacrylates). These gels do not require the use of an additional crosslinking agent, but produce heterogeneous networks structures that contain dense crosslinking regions (Lin and Anseth, 2009b; Van Hove et al., 2013). Step-growth polymerization occurs when PEG macromers preferentially react with a second functionality on a crosslinker (thiol-acrylate, thiol-norbornene, alkyne-azide, tetrazine-azide, etc.). Step-growth polymerization provides an easy method to incorporate peptides into hydrogel networks; by exploiting thiol groups on cysteine amino acids and unsaturated carbon bonds of functionalities introduced to PEG (norbornene, acrylate, etc.), peptides can be incorporated into hydrogels as crosslinking agents or tethered pendant groups (Fairbanks et al., 2009; Shih and Lin, 2012; Van Hove et al., 2014).

###### PEG hydrogels for pro-angiogenic drug delivery

Poly(ethylene glycol) hydrogels have been used to deliver a number of therapeutic molecules. This specific topic was previously reviewed by Lin and Anseth (2009b). Herein, we will focus on recent developments and their specific utility in pro-angiogenic applications. Controlled delivery of human growth hormone (hGH) from step-growth polymerized PEG hydrogel networks formed by reacting multi-arm PEG acrylate (PEGA) with dithiothreitol (DTT) has been demonstrated. Precipitation of hGH with Zn^2+^ prior to encapsulation protected the protein during polymerization and delayed release from the gels via delayed dissolution (Figure 2E). Varying the PEG macromers used to form hydrogels controlled hydrogel swelling ratios, which subsequently extended protein release beyond 25 days (van de Wetering et al., 2005). While all gels studied were hydrolytically degradable, only one macromer configuration produced gels that degraded and release drug over similar time frames (21 days). The other gels developed persisted long after releasing their payload: gels releasing hGH over 1 day took 21 days to degrade, and gels releasing hGH over ~8 weeks (extrapolated based on first-order release data) were still intact after 15 weeks (van de Wetering et al., 2005). hGH has been shown to have both pro- and anti-angiogenic effects depending if it is presented in full-length protein or as the 16 kDa N-terminal fragment (Struman et al., 1999). Therefore, while these hydrogels were intended for treatment of growth hormone deficiency, Turner’s syndrome, and chronic renal failure, they could also present a promising pro-angiogenic strategy. While to the best of our knowledge, this delayed dissolution approach has not yet been exploited for delivery of peptide drugs, it could theoretically be used for delivery of hydrophobic peptides, such as Qk, which has 53% hydrophobic amino acids (Lehninger et al., 2000) and a Hopp–Woods average value of −0.2 (Hopp and Woods, 1981). Additionally, Qk forms an α-helix, which has been shown to increase peptide self-assembly in aqueous solution (Kisiday et al., 2002), making it an attractive candidate for delivery using this delayed dissolution approach.

poly(ethylene glycol) hydrogels designed by West and Hubbell to degrade in response to local enzyme levels have recently been adapted for drug delivery applications. In their seminal work, enzymatically responsive PEG macromers were formed by reacting a degradable peptide with PEG, forming an peptide-PEG-peptide block copolymer which was then functionalized with terminal acrylate groups, allowing for hydrogel formation (West and Hubbell, 1999). Building upon this foundation, additional enzymatically responsive PEG hydrogels have been developed, with the specific degradable peptide used controlling enzyme specific and hydrogel degradation kinetics (Hubbell et al., 2003; Patterson and Hubbell, 2010).

As a pro-angiogenic approach, Zisch et al. used Michael-type addition reactions to form PEG hydrogels that degrade and release VEGF in response to local enzymes. Multi-arm PEG vinyl sulfone was reacted with cysteine flanked MMP-degradable peptides, as well as VEGF engineered with a plasmin-sensitive tether and terminal cysteine. This formed hydrogels that released VEGF both upon MMP-mediated hydrogel degradation and plasmin-mediated tether cleavage (Figures 2B,C). When used in the chick chorioallantoic membrane assay, VEGF-conjugated hydrogels resulted in the formation of new vessels highly localized to the hydrogel, and improved vessel infiltration upon subcutaneous implantation as compared to controls (Zisch et al., 2003). These hydrogels simultaneously degrade and release the pro-angiogenic protein, attractive behavior for an implantable or injectable drug delivery system as the gel will not persist after delivering its payload. This system could be easily adapted for delivery of pro-angiogenic peptides like those shown in Table 2. However, some of the drug is release tethered to a PEG molecule. While testing showed that VEGF remained bioactive with the PEG “tail” (Zisch et al., 2003), this would not necessarily be the case for all proteins or peptides.

Phelps et al. also exploited PEG hydrogels to provide enzymatically responsive protein release. By reacting proteolytically cleavable peptide linkers with acrylate-PEG-*N*-hydroxysuccinimide (NHS), an acrylate–PEG-peptide–PEG-acrylate macromer was formed. These macromers were then polymerized to form enzymatically degradable PEG hydrogels, with VEGF tethered via a non-degradable PEG linker, resulting in protein release only when the gel is degraded (Figure 2B). Similar to the system developed by Zisch et al., the VEGF is released from the gel tethered to residual PEG macromers. While enzymatically responsive hydrogel degradation was shown, protein release was not quantified in parallel. Nevertheless, there was significantly greater vascular ingrowth into VEGF-releasing hydrogel as compared to the enzymatically responsive hydrogels alone. Additionally, treatment with the enzymatically responsive, VEGF-releasing hydrogels caused greater reperfusion of ischemic hindlimb tissue than bolus VEGF delivery (Phelps et al., 2010).

Our group recently developed hydrogels providing sustained, enzymatically responsive peptide release (Figure 2B). Peptide drugs were synthesized flanked by enzymatically degradable sequences with terminal cysteine amino acids (C-degradable linker-drug-degradable linker-C). This allowed for step-growth thiolene reactions with multi-arm norbornene-functionalized PEG (PEGN). These hydrogels demonstrated enzymatically responsive degradation and peptide release, and were confirmed to release bioactive components able to induce tube network formation *in vitro*. Similar to the previously discussed systems, simultaneous hydrogel degradation and peptide release occurs. However, in this system, the peptide drugs are released with only four amino acids residues on either side of the drug, rather than entire PEG macromers. These residual amino acids still had a substantial effect of peptide bioactivity, with only three of the six pro-angiogenic peptides screened retaining bioactivity *in vitro*. While this work generated a novel biomaterial to provide enzymatically responsive delivery of peptide drugs and identified key drug properties that affect gel behavior, it did not investigate hydrogel pro-angiogenic efficacy *in vivo* (Van Hove et al., 2014).

pH and temperature-responsive materials have also been exploited for controlled delivery of pro-angiogenic factors. Temperature and pH-responsive copolymers were formed from *N*-isopropylacrylamide (NIPAAM), propylacrylic acid (PAA), and butyl acrylate (BA) monomers [p(NIPAAm-co-PAA-co-BA)]. These copolymers form physical hydrogels (undergoing solution-to-gel, or sol-to-gel, transition) as temperature is increased and pH decreased, with the transition point affected by the relative amounts of each monomer used. p(NIPAAm-co-PAA) (83 mol% NIPAAm, 17 mol% PAA, 37 kDa) copolymers produced hydrogels that released encapsulated VEGF via diffusion over ~7 days at pH 7.4 and over ~3 weeks when pH was lowered to 5 or 6, pH levels consistent with ischemic tissue microenvironments (Garbern et al., 2010). Similar p(NIPAAm-co-PAA-co-BA) copolymers (67 mol% NIPAAm, 18 mol% PAA, 15 mol% BA, 28 kDa) delivered encapsulated bFGF *in vivo* to infarcted myocardium over ~7 days, and improved fractional shortening of and blood flow to the heart, as well as capillary and arteriolar densities as compared to controls (Murry et al., 2011). pH-responsive nanospheres formed from p(pAA-PEG) have been used to provide stimuli-responsive release of insulin (Foss et al., 2004), demonstrating the potential of these similar pH-responsive hydrogels for the delivery of pro-angiogenic peptide drugs.

##### Hybrid materials

Combinations of natural and synthetic polymers have also been used to form hybrid hydrogels for controlled drug delivery. Heparin is often exploited as a natural polymer, as many pro-angiogenic proteins contain heparin-binding domains. Via heparin-protein affinity interactions, protein release can be sustained for days to weeks, depending upon the protein, amount of heparin included, and the tissue microenvironment. For example, covalently crosslinked heparin-PEG gels were formed by step-growth reactions between hydrazide-functionalized heparin (Hep-ADH) and poly(ethylene glycol)-bis-butanoic acid (SBA-PEG-SBA). After gel formation, gels were partially dried and injected with a high concentration of VEGF before being incubated overnight to allow the protein to equilibrate within the gel. Release of VEGF from these hydrogels was nearly linear and occurred over >3 weeks, with the extended release attributed to affinity interactions between VEGF and heparin (Figure 2D). Subcutaneous implantation of the hydrogels showed increased CD31-staining as compared to control gels, indicating increased vessel formation (Tae et al., 2006).

Numerous proteins beyond VEGF contain heparin-binding domains (Peysselon and Ricard-Blum, 2014), and as a result, heparin affinity has been exploited for controlled delivery of a variety of proteins from a number of materials. This includes delivery of bFGF and BMP-2 from PEG hydrogels (Benoit and Anseth, 2005; Benoit et al., 2007; Nie et al., 2007), bFGF and β-nerve growth factor (β-NGF) from fibrin hydrogels (Sakiyama-Elbert and Hubbell, 2000a,b), and transforming growth factor β1 (TGF-β1), FGF-2, VEGF, and BMP-2 from alginate hydrogels (Jeon et al., 2011), all which incorporated heparin functionalities to prolong growth factor delivery. These materials are well suited for delivery of pro-angiogenic peptides, such as PAB2-1c, which contains the heparin-binding region RKRKLERIAR (Lin et al., 2007). Additional pro-angiogenic peptides could be delivered from heparin-functionalized hydrogels by including this or another heparin-binding sequence (Verrecchio et al., 2000) on either the C- or N-termini of the drug sequence, provided the addition of the heparin-binding region did not inhibit peptide bioactivity. However, as previously discussed, materials exploiting heparin affinity have the potential to sequester a variety of host proteins with affinity for heparin (Peysselon and Ricard-Blum, 2014).

Similarly, short peptides have been identified that mimic the heparin-binding capacity of VEGF (Maynard and Hubbell, 2005) and NGF (Willerth et al., 2007). By conjugating these peptides to hydrogels, affinity-controlled release of NGF from fibrin hydrogels (Willerth et al., 2007) and bFGF from PEG hydrogels (Lin and Anseth, 2009a) was demonstrated. While not explicitly used for pro-angiogenic applications, the potential for these approaches to deliver a number of pro-angiogenic proteins make them promising materials that could be further exploited for pro-angiogenic applications. However, these binding peptides would not necessarily have the same ability to bind and control the release of peptides mimics, as these mimics do not necessarily contain the region responsible for the drug-binding peptide interaction.

Other hybrid materials approaches for pro-angiogenic factor release include step-growth PEG-dextran hydrogels. These networks were formed by Michael Addition reactions between dextran vinyl sulfone (dex-VS) and multi-arm PEG thiol. Controlling the molecular weight of the dextran molecule and the degree of substitution was shown to control hydrogel degradation kinetics and delivery of encapsulated proteins, such as immunoglobulin G (IgG) and bovine serum albumin (BSA). Lysozyme and bFGF release was achieved over 2 weeks to 1 month, with release rates affected by hydrogel composition. While the ability of the bFGF-releasing hydrogels to induce angiogenesis was not studied *in vivo*, this material successfully delivered the pro-angiogenic factor over 28 days with first-order release kinetics, with hydrogel degradation occurring over a similar time scale (Hiemstra et al., 2007). These dextran-PEG hydrogels release encapsulated protein by hindered diffusion (Figure 2A), and would likely release peptides at an accelerated rate due to the smaller size of the peptide drugs. However, it is possible that extended release could be achieved by further increasing the degree of substitution on the dextran or decreasing the molecular weight, both of which prolonged the protein delivery (Hiemstra et al., 2007).

Hybrid scaffolds have been formed using semi-interpenetrating polymeric network (semi-IPN) of poly(ether)urethane-polydimethylsiloxane (PEtU-PDMS) networks coated with protein-laden fibrin gels. These materials combined the mechanical strength of the PEtU-PDMS scaffold with controlled release provided by fibrin gels. This combination material provided simultaneous release of bFGF and VEGF, and significantly improved capillary density and perfusion of ischemic murine hind limbs as compared to controls (Losi et al., 2010). However, these materials must be formed *ex vivo*, and the PEtU-PDMS scaffold persists over a longer time scale (~6–24 months) (Soldani et al., 2010) than they deliver the drug (~1–2 weeks) (Losi et al., 2010). These scaffolds relied on diffusional release of the protein from the fibrin gels, rather than covalently linking the protein to the gel as discussed in the Section “Fibrin.” This makes them an unattractive approach for delivery of pro-angiogenic peptides, as the release of the drug is governed by diffusion (Figure 2A) rather than degradation, and would likely result in accelerated release of pro-angiogenic peptides. Alternately, one could envision combining the enzymatically responsive fibrin material used by Zisch et al. and Ehrbar et al. with the PDMS scaffold exploited here to combine the benefits of prolonged, enzymatically responsive drug delivery with the strength of the PDMS scaffold (Zisch et al., 2001; Ehrbar et al., 2004, 2005).

Many HA-based hydrogels are crosslinked by functionalizing HA and PEG with mutually reactive groups (Peattie et al., 2004; Cai et al., 2005; Pike et al., 2006; Riley et al., 2006; Hosack et al., 2008). Hydrogels have been formed by reacting thiol-functionalized HA with PEG diacrylate (Peattie et al., 2006; Pike et al., 2006; Riley et al., 2006; Hosack et al., 2008), hydrazide-functionalized HA with PEG propiondialdehyde (Peattie et al., 2004), and methacrylate-functionalized HA with PEGA (Leach and Schmidt, 2005). HA hydrogels containing tethered gelatin and heparin were formed containing encapsulated VEGF or bFGF, and showed extended release of encapsulated protein, with tunable release varying from 19 to 96% after 42 days. Inclusion of thiol-functionalized gelatin increased the rate of protein release, while increasing amounts of heparin decreased the rate of drug delivery, with similar trends observed for both VEGF and bFGF. Heparin-functionalized HA gels releasing VEGF and bFGF both increased vascularization index 28 days after implantation in the mouse ear, but vessel density was unaffected (Pike et al., 2006). Excitingly, HA hydrogels crosslinked with PEG releasing encapsulated VEGF and/or keratinocyte growth factor (KGF) showed an additive increase in vessel number after implantation in the mouse ear when compared to HA gels, VEGF, or KGF alone. However, this study did not report the rate of drug release or degradation of the material (Peattie et al., 2006). These hydrogels could potentially be adapted for peptide delivery, but would require similar modification of peptides with a heparin-binding region as discussed above, to facilitate extended release.

#### Scaffold-Based Systems

Many studies have exploited poly(lactide-co-glycolide) (PLG) scaffolds for drug delivery applications. PLG materials are biodegradable, biocompatible, and have received FDA approval for drug delivery applications (Jain, 2000; Makadia and Siegel, 2011). By altering the relative amounts of poly(lactic acid) (PLA) and poly(glycolic acid) (PGA) in the copolymer, the rate of degradation and subsequent release of encapsulated drug can be controlled. While commonly used to form nano- and microparticles for systemic drug delivery, PLG can also be fabricated into scaffolds for depot-based drug delivery. One important considerations for use of PLG scaffolds is that the rate of degradation and associated drug release is dependent on many factors (lactide/glycolide ratio, polymer molecular weight, degree of crystallinity, glass transition temperature, etc.) and can be difficult to predict (Makadia and Siegel, 2011). Additionally, degradation of PLG scaffolds releases lactic and glycolic acid, which can accelerate the rate of degradation and affect local tissue pH, potentially damaging nearby tissue (Liu et al., 2006). Select examples of scaffolds used for controlled drug delivery are listed in Table 4.

**Table 4:** **Scaffold-based biomaterials for controlled drug delivery**.

Polymer	Drug Delivered	Drug Type	Mode of release	Reference
Poly(lactide-co-glycolide) (PLG)	VEGF	Protein	Scaffold degradation	Sun et al. (2005)
PLG	VEGF and PDGF	Dual proteins	Diffusion and scaffold degradation	Mooney et al. (2007)
PLG	VEGF and PDGF	Dual proteins	Diffusion and scaffold degradation	Richardson et al. (2001)
PLG	VEGF/Ang2 and PDGF/Ang1	Multiple proteins	Diffusive and scaffold degradation	Brudno et al. (2013)

*Select examples of scaffold-based biomaterials used for controlled drug delivery*.

For pro-angiogenic applications, PLG scaffolds have been developed using a high-pressure carbon dioxide/salt leaching method where the delivery of VEGF is controlled by the rate of scaffold degradation (Figure 2F). Sustained VEGF delivery over ~1 month was achieved, with the PLG–VEGF scaffold significantly increasing reperfusion of, and capillary density within, ischemic murine hindlimb as compared to empty scaffolds (Sun et al., 2005). In an attempt to mimic the temporal growth factor expression occurring in healthy tissue (Figure 1), PLG scaffolds were formed releasing VEGF followed by PDGF. In layer 1, PDGF was pre-encapsulated in PLG microspheres and mixed with VEGF prior to scaffold formation, while layer 2 only contained VEGF. This resulted in spatially patterned scaffolds that provided delayed release of PDGF compared to VEGF. These dual-delivery scaffolds induced the formation of more, larger vessels than empty PLG scaffolds. However, due to the spatial patterning of the two layers, local protein delivery to the tissue was dependent on scaffold orientation during implantation (Mooney et al., 2007).

Alternately, by forming PLG scaffolds with microspheres already containing PDGF, which are subsequently mixed with VEGF, sustained release of both factors was achieved, with VEGF being released more rapidly than PDGF. Similar to the system just discussed, this biomaterial attempts to mimic the native pro-angiogenic signaling cascade (Figure 1); however, this system has the advantage of spatial uniformity, causing temporal protein delivery to be independent of scaffold orientation. This dual-growth factor delivery system increased vessel density within the scaffold after subcutaneous implantation compared to scaffolds delivering either factor alone. Dual factor delivery also increased vessel size and maturity as compared to blank scaffolds or scaffolds releasing VEGF or PDGF alone (Richardson et al., 2001). This biomaterials strategy has also been used to temporally control the delivery of multiple pro-angiogenic (VEGF and Ang2) and pro-maturation (PDGF and Ang1) factors from a single scaffold. Scaffolds delivering all four growth factors over time courses motivated by healthy angiogenic signaling (Figure 1) and *in vitro* testing resulted in the formation of more mature vessels than controls and the formation of the largest vessels of any group investigated (Brudno et al., 2013). These PLG scaffold systems could easily be adapted for controlled delivery of peptide drugs, as the drugs (protein or peptide) are released upon degradation of the biomaterial and do not rely on size-hindered diffusion through the material (Figure 2F). Similar to the results showing improved vascularization upon co-delivery of VEGF and PDGF, PLG scaffolds could be developed that deliver Qk (the VEGF mimic) followed by PAB2-1c (the PDGF mimic) or T7 (Ang1 mimic).

## Engineering Successful Pro-Angiogenic Biomaterials

While a number of pro-angiogenic biomaterials have been discussed here, they are not equally well-suited to all pro-angiogenic therapeutic applications. All materials meet the over-arching requirement of biocompatibility, but the different environments and demands of ischemic peripheral tissue, ischemic cardiac tissue, diabetic ulcers, and tissue engineering applications result in vastly different material requirements. Ischemic cardiac tissue, for example, is most likely to be successfully treated by injectable materials that can be delivered laparoscopically, to provide initial structural support to the damaged heart, produce extended growth factor release, and eventually degrade (Chen et al., 2008). These design requirements makes the alginate, fibrin, ECM-based, enzymatically degradable PEG, and PEG-dextran gels discussed here very promising for cardiac applications, as they can be crosslinked *in situ*, either using a dual barrel syringe or UV-initiated photopolymerizations. For all materials, testing would be required to ensure *in situ* polymerization produces gels that provide similar drug release and vascularization as *ex situ* polymerization, to address potential differences in crosslinking efficiency and drug encapsulation that could occur. The pH and temperature-responsive p(NIPAAm-co-PAA-co-BA) hydrogels have the added benefit of being crosslinked by the native tissue environment, thus avoiding the need for external stimuli for gelation (Garbern et al., 2010; Murry et al., 2011). Ischemic peripheral tissue has similar design requirements as cardiac applications and would likely be best treated by the same types of materials as cardiac tissue. While the use of biomaterials for cardiac regeneration has largely focused on delivery of pro-angiogenic proteins, multiple pro-angiogenic peptides could instead be delivered to ischemic cardiac tissue, such as Qk, AcSDKP, and T7, all of which have shown promising results in other *in vivo* models (Liu et al., 2003; Santulli et al., 2009; Slyke, 2011).

Diabetic wounds are readily accessible, and therefore do not require *in situ* gel formation or degradation, as the biomaterial can simply be placed on the wound and removed after delivering its payload. Therefore, these ulcers could be treated by a wider range of materials, including the gelatin and PEtU-PDMS materials discussed here. Diabetic ulcers are associated with myriad deficiencies beyond insufficient angiogenesis (Lobmann et al., 2002; Lerman et al., 2003; Galiano et al., 2004), and as such, would likely benefit from delivery of multiple protein or peptide drugs, or drugs that have more broad effects, such as GHK-containing peptides (Pickart, 2008). Additionally, some of the pro-angiogenic peptides discussed here have already been shown efficacious in treating diabetic wounds, such as T7 and GHK, making them even more attractive candidates for incorporation into biomaterials for treatment of these wounds (Pickart, 2008; Van Slyke et al., 2009). As PLG scaffolds must be formed externally and then implanted, they could also be used for diabetic ulcer treatment, but are not ideal for cardiac applications. However, these scaffolds present a unique material compared to the other gels discussed here, in that they have significantly greater structural integrity (Makadia and Siegel, 2011). This makes them the most attractive biomaterial discussed here for engineering tissues, such as bone, while softer gels are better suited for engineering more compliant tissues, such as kidney and liver. However, as previously discussed, the acidic environment caused by degradation of the PLG scaffolds can negatively affect tissue, and must be considered when using these biomaterials (Liu et al., 2006).

## Concluding Remarks

From natural to synthetic, diffusion controlled to stimuli-responsive, a number of biomaterials delivery systems have been developed to deliver pro-angiogenic factors, each presenting unique advantages and disadvantages. Building off seminal research, current research is producing more complex and intricate materials delivering pro-angiogenic drugs, inducing robust pro-angiogenic effects *in vivo*. Delivery of multiple factors, with tight temporal control over factor release has been shown to induce formation of more mature vasculature than delivery of a single factor. Similarly, materials delivering pro-angiogenic factors in response to enzyme expression present a promising means to deliver drugs based on local tissue demands. While current research focuses largely on delivery of pro-angiogenic proteins, we feel that delivery of peptide drugs that mimic the bioactivity of these proteins presents a unique opportunity to develop novel, potent pro-angiogenic therapies. Additionally, materials combining many of the promising techniques already developed could present even more potent methods to induce therapeutic angiogenesis, such as temporally controlling stimuli-responsive release, or delivery of multiple factors in a stimuli-responsive manner.

## Author Contributions

Literature review was performed by AVH with assistance by DB. AVH generated all figures and tables, and wrote the article. AVH and DB critically revised the work and approved the final version to be published.

## Conflict of Interest Statement

The authors declare that they have no financial conflicts of interest related to the submitted work. They have a patent pending on one of the pro-angiogenic biomaterial discussed (Amy H. Van Hove and Danielle S. W. Benoit. Compositions and Methods for Stimuli-Responsive Release of a Therapeutic Agent. PCT/US14/49774 (filed). Assignee: University of Rochester. 5 August 2014.)
